# Supplementary material for: Strong band renormalization and emergent ferromagnetism induced by electron-antiferromagnetic-magnon coupling
Source: Nat Commun. 2022 Nov 2;13:6560. doi: 10.1038/s41467-022-34254-0 (PMC9630309; doi:10.1038/s41467-022-34254-0)
Supplement: Supplementary file 1 — Supplementary Information [file 41467_2022_34254_MOESM1_ESM.pdf]

Supplemental Materials for

Strong Band Renormalization and Emergent Ferromagnetism induced by Electron-Antiferromagnetic-Magnon Coupling

T. L. Yu,[1] M. Xu,[1] W. T. Yang,[1] Y. H. Song,[1] C. H. P. Wen,[1] Q. Yao,[1] X. Lou,[1] T. Zhang,[1] W. Li,[1] X. Y. Wei,[1] J. K. Bao,[4] G. H. Cao,[4] P. Dudin,[5] J. D. Denlinger,[6] V. N. Strocov,[7] R. Peng,[1, 2, *] H. C. Xu,[1, †] and D. L. Feng[2, 3, 8, ‡]

[1] *Laboratory of Advanced Materials, State Key Laboratory of Surface Physics and Department of Physics, Fudan University, Shanghai 200438, People's Republic of China*

[2] *Shanghai Research Center for Quantum Sciences, Shanghai 201315, People's Republic of China*

[3] *Collaborative Innovation Center of Advanced Microstructures, Nanjing 210093, China*

[4] *Department of Physics, Zhejiang University, Hangzhou 310027, People's Republic of China*

[5] *Diamond Light Source, Harwell Science and Innovation Campus, Didcot OX11 0DE, United Kingdom*

[6] *Advanced Light Source, 1 Cyclotron Road Lawrence Berkeley National Laboratory Berkeley, CA 94720-8229, USA*

[7] *Swiss Light Source, Paul Scherrer Institut, CH-5232 Villigen PSI, Switzerland*

[8] *Hefei National Laboratory for Physical Science at Microscale, CAS Center for Excellence in Quantum Information and Quantum Physics and Department of Physics, University of Science and Technology of China, Hefei 230026*

1. Bulk nature of the measured band structure

The bulk nature of the measured band structure of $Ba_{1-x}K_xMn_2As_2$ is confirmed by soft X-ray ARPES measurement. Due to the surface sensitivity of VUV ARPES, the photoemission signal sometimes could be dominated by surface states in certain material systems. The soft X-ray ARPES technique is more bulk sensitive because of the larger escaping depth of photoelectrons excited by soft X-ray photons. The in-plane and out-of-plane electronic structure of $Ba_{1-x}K_xMn_2As_2$ (x = 0.3) measured by soft X-ray ARPES (Fig. S1) agrees well with those measured by VUV ARPES in Fig. 1 of the main text. This ensures that the electronic structure from VUV ARPES also represents the intrinsic bulk behavior. Note that these data were taken under C^+ polarization. Although the matrix elements effect is reduced under soft X-ray energy range, the C^+ polarization causes an anisotropic result naturally, which is enhanced in the neighbor BZs in our results. Moreover, the intensity image is a bit distorted near the edge of electron lens. These two facts make the Fermi surface looks a bit different in the neighbor BZs, but do not affect our conclusions.

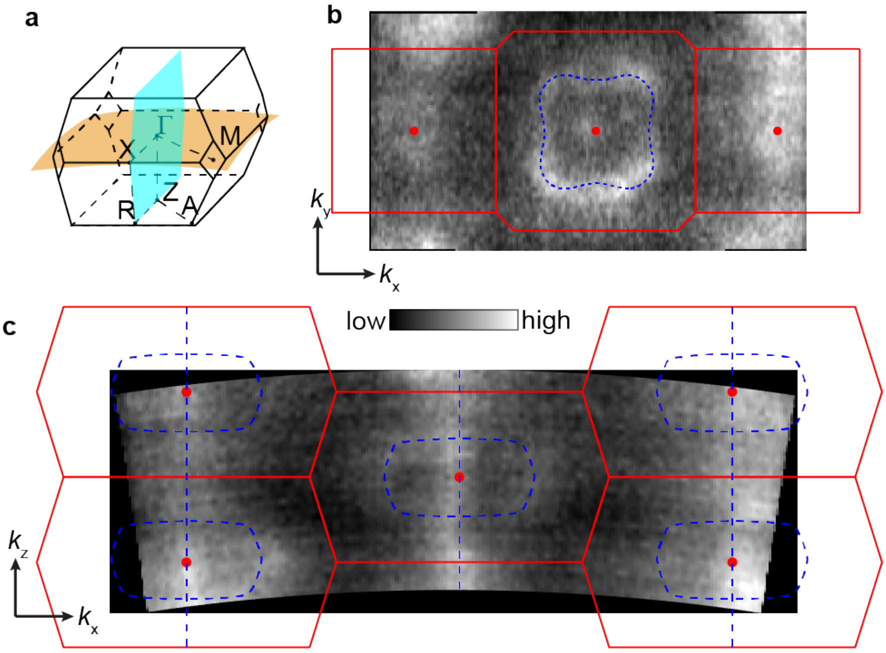

Supplementary Figure S1. Three-dimensional Fermi surface of $Ba_{1-x}K_xMn_2As_2$ (x = 0.3) from soft X-ray ARPES measurements. (a) Three-dimensional Brillouin Zone of $Ba_{1-x}K_xMn_2As_2$. (b) Photoemission intensity map near the ΓXM plane measured by 565 eV photons of C^+ polarization. (c) Photon energy dependent photoemission intensity map in the ΓZR plane using photons from 506 eV to 620 eV. The Fermi surface maps are integrated over an energy window of $E_F \pm 50$ meV. The blue dashed curves indicate the Fermi surface pockets of band α and the intensity from bands β/β'. The data were measured at 26 K at ADRESS beamline of Swiss Light Source.

2. Doping dependence of the Fermi surface volumes

The Fermi surface volume of all bands crossing E_F has been calculated to obtain the carrier doping level. Only band α crosses E_F for $x = 0.1$ and 0.2, while bands α, β and β' all cross E_F for $x = 0.3$ (Fig. S2a-c). The 3-dimensional character of Fermi surfaces have been considered when calculating the Luttinger volume. As stated in the main text, band α forms a 3D drum-shaped Fermi surface while bands β and β' forms two elliptical cylinders. We estimated the Fermi surface volume of band α using a truncated hexahedron scaled to the experimental Fermi surface. The uncertainty of the Luttinger count is estimated to be 15% by $\frac{\Delta V}{V} = \sqrt{3\left(\frac{\Delta k_{\mathrm{F}}}{k_{\mathrm{F}}}\right)^2 + \left[\frac{(k_{\mathrm{F}}^{max})^3-(k_{\mathrm{F}}^{min})^3}{3((k_{\mathrm{F}}^{max}+k_{\mathrm{F}}^{min})/2)^3}\right]^2}$,

where k_F^{min} stands for the minimum possible size of the truncated hexahedron, k_F^{max} the maximum possible size of the truncated hexahedron, Δk_{F} the uncertainty of k_F from fitting MDCs. The dopings calculated from Fermi surfaces (Fig. S2e) based on Luttinger theorem agree well with the chemical doping determined by EPMA measurements, following the behavior of a normal metal.

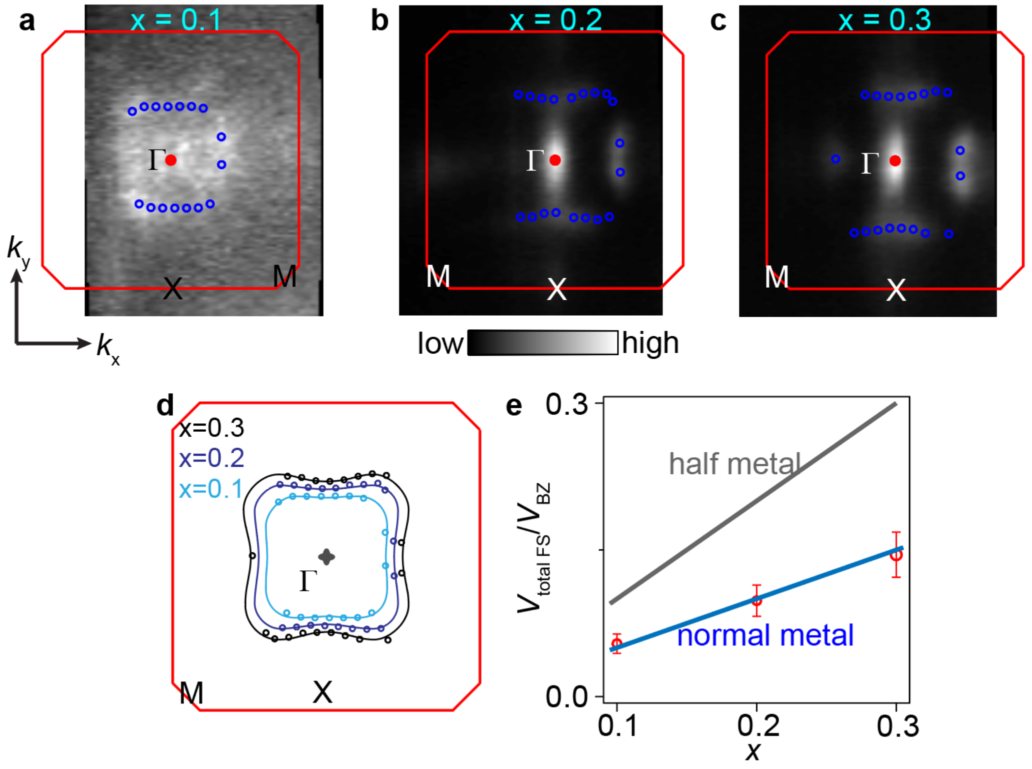

Supplementary Figure S2. Fermi surface maps for different dopings: $x = 0.1$ (a), 0.2 (b) and 0.3 (c). The Fermi surface maps are integrated over an energy window of $E_F \pm 20$ meV. The circles track the local maxima of MDCs at E_F for the Fermi crossings. The data were measured using 78 eV photons (corresponding to ΓXM plane) at 30K at SSRL. (d) Summary of the Fermi surfaces at different dopings. (e) Normalized total Fermi surface volume as a function of chemical dopings. The grey and blue curves illustrate the expected doping dependencies of normal metal and half metal, respectively. The data were measured at 30 K, BL5-2 of SSRL.

3. The homogeneity of K doping and metallic phase in $Ba_{1-x}K_xMn_2As_2$

It is well known that many K-relevant compounds suffer from inhomogeneity and phase separation, such as KFe_2Se_2 and $Ba_{1-x}K_xFe_2As_2$. Thus, it is important to prove that our data of $Ba_{1-x}K_xMn_2As_2$ is from homogeneous samples and reflects the intrinsic properties. The homogeneity of K doping and metallic phase in $Ba_{1-x}K_xMn_2As_2$ is justified by the following experimental facts:

1) The dopings calculated from Fermi surfaces (Fig. S2e) based on Luttinger theorem agree well with the chemical doping determined by EPMA measurements, demonstrating that most of the sample is single crystalline $Ba_{1-x}K_xMn_2As_2$ with homogenous potassium doping.
2) If there were undoped $BaMn_2As_2$ phase that hypothetically dominates the neutron scattering signal and shows robust T_N and magnetic moment μ_B, its volume and mass must also dominate the sample. The undoped $BaMn_2As_2$ is an AFM insulator with a band gap, whose ARPES spectra has been reported (Figs. 1b-c in Ref. 1). Compared with the results in ref. 1, only the band structure from doped $Ba_{1-x}K_xMn_2As_2$ phase is observed in our data (Fig. S3), thus single crystalline undoped $BaMn_2As_2$ phase is negligible, if any.
3) We performed STM measurements on the same sample in ARPES experiment with $x = 0.2$ on a Createc System. Fig. S4a shows a typical topography image of the cleaved surface of $Ba_{1-x}K_xMn_2As_2$. Across the 800nm×800nm field of view, the STS dI/dV spectra show consistent metallic behavior with similar line shape (Fig. S4b). Phase separation of insulating and metallic regions is not observed. In the 20nm×20nm area, the dopant atoms are randomly distributed (Fig. S4c), which is typical for BaK terminated surfaces like those in $Ba_{1-x}K_xFe_2As_2$ with clear superconducting gap (Fig. 1a in Ref. 2). These results are repeatable over the sample surface of millimeter size. These observations demonstrate that the metallic behavior and chemical doping are homogenous over the entire sample.
4) In ref. 3, the MnO_2 impurity is less than 0.6wt% in the polycrystal sample $Ba_{1-x}K_xMn_2As_2$ x=0.25. It should be even less in the single crystalline sample. Such a small amount of MnO_2, if any, would be insulating, which will not contribute to the photoemission signal near Fermi energy. Even if the amount of impurities is large, it could lead to imperfections in single crystals, resulting in energy-independent momentum broadening of the electronic spectra rather than a kink feature. Therefore, the observed kink feature in $Ba_{1-x}K_xMn_2As_2$ is intrinsic from the homogenously doped metallic phase.
5) Electron probe micro-analysis (EPMA) measurement on cleaved surface of $Ba_{1-x}K_xMn_2As_2$ have been performed in a 5×5 mesh of 20 μm × 20 μm area (Fig. S5a) and 4 μm × 4 μm area (Fig. S5b). The histogram of doping value x (Fig. S5c) indicates homogeneous chemical doping of K in the measured length scale.

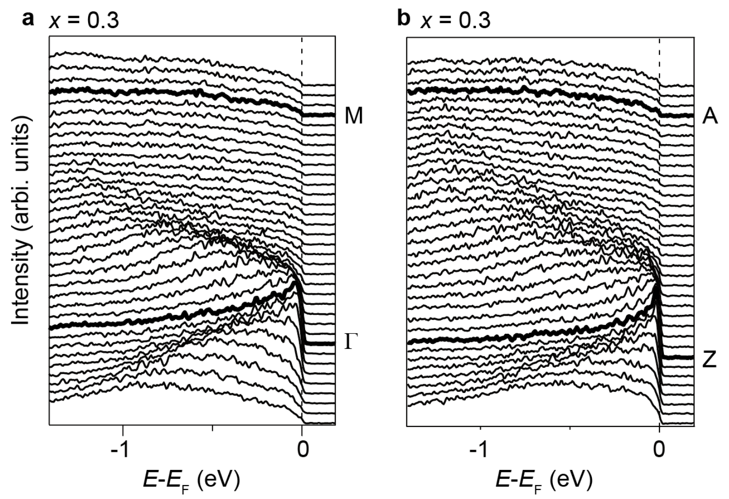

Supplementary Figure S3. (a-b) ARPES spectra at the same momentum and energy range from our $Ba_{1-x}K_xMn_2As_2$ x=0.3 sample.

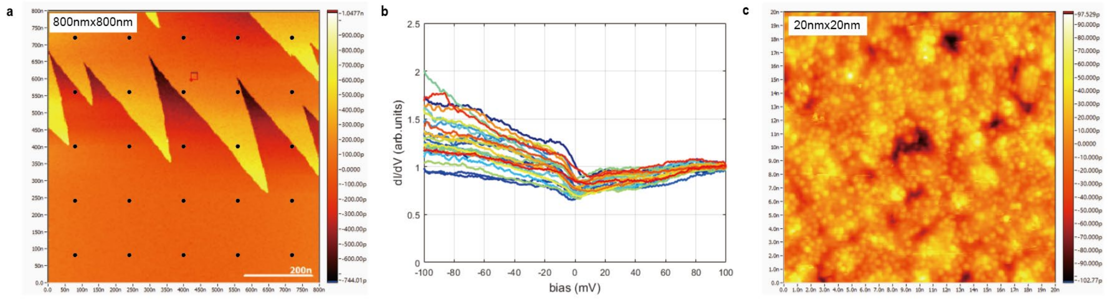

Supplementary Figure S4. (a) Typical STM topography on the cleaved surface of $Ba_{1-x}K_xMn_2As_2$ x=0.2 in an 800nm×800nm area. (b) STS dI/dV curve at the black dots in panel a. (c) STM topography in a 20nm×20nm area as illustrated by the red square in panel a.

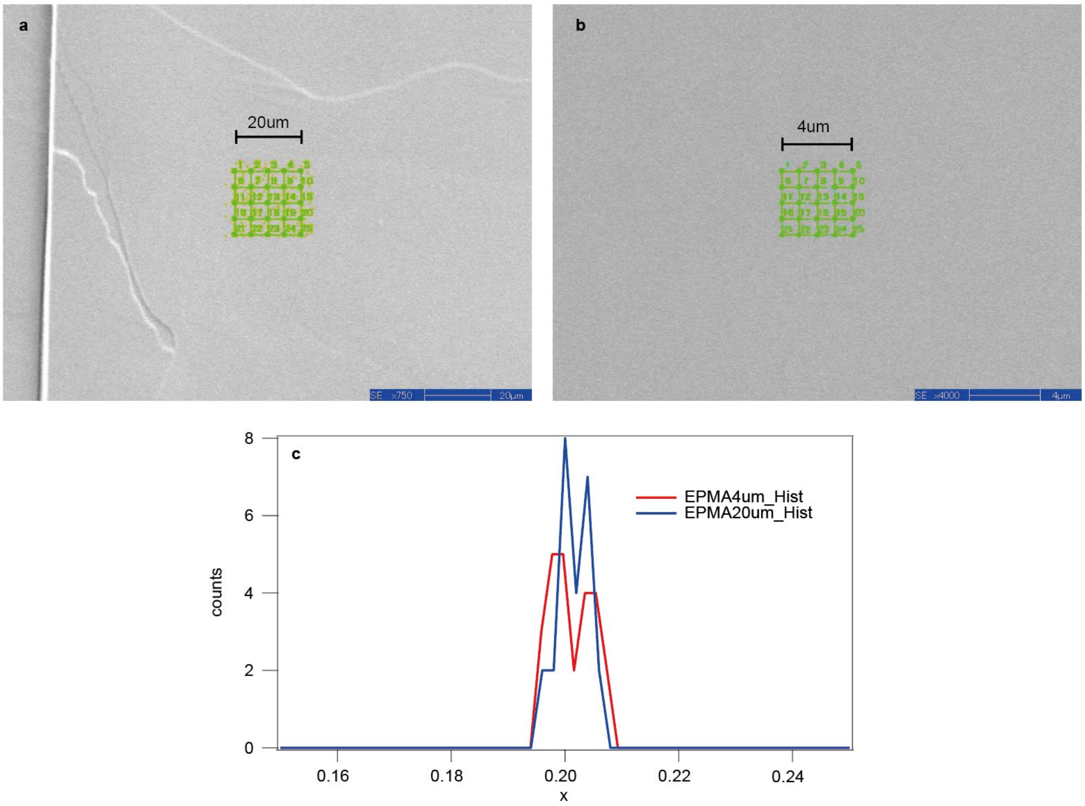

Supplementary Figure S5. (a) Scanning electron microscopy (SEM) picture of the cleaved surface of $Ba_{1-x}K_xMn_2As_2$ x=0.2 and the illustration of a 5×5 mesh of 20 μm × 20 μm area. (b) Same as panel a but for illustrating a 5×5 mesh of 4 μm×4 μm area. (c) Histogram of the chemical dopings x measured at the points of the meshes in panels a and b. The histogram analysis is performed using window width of Δx=0.02.

4. ARPES spectra at different k_z for x = 0.3

The band α shows kink feature around E_B=50 meV and single-branch dispersion for different photon energies (Fig. S6). The data show no separate band formation, while the kink feature is always observable. This excludes the alternative interpretation of two branches of bands.

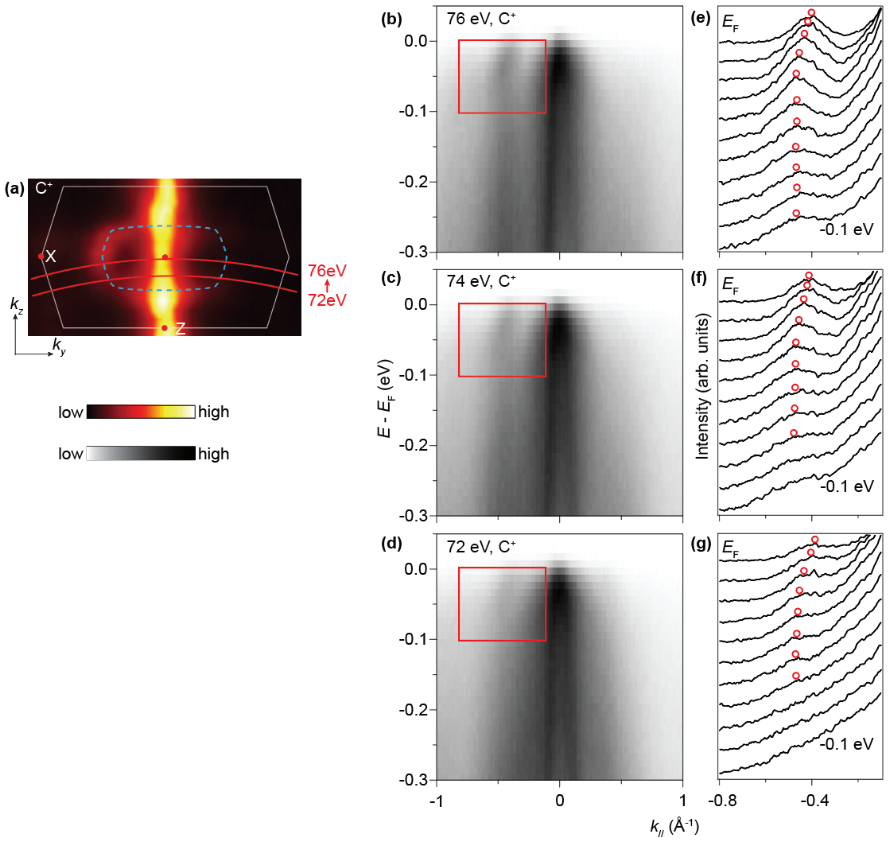

Supplementary Figure S6. (a) Photoemission intensity map in the k_y-k_z plane, where the red curves illustrate the momentum cuts corresponding to 72eV and 76eV photon energies. (b)-(d) Photoemission spectra taken with photon energies 76eV, 74eV, and 72eV, respectively. (e)-(g) MDCs around the kink feature of band α, corresponding to the region illustrated by red rectangles in panels b-d. The red circles trace the dispersion of band α. The data were measured at 30K, BL5-2 of SSRL.

5. The near-E_F dispersion and kink feature in band β

The photoemission data of ferromagnetic $Ba_{1-x}K_xMn_2As_2$ (x = 0.3) shows a kink feature in the dispersion of band β around 50 meV. However, it is hard to extract the self-energy near the band top where two branches of band β merge with each other.

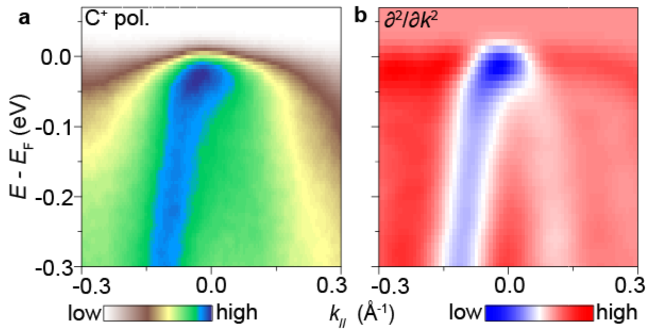

Supplementary Figure S7. (a) Symmetrized photoemission intensity data along Γ-X obtained by s-pol. (b) The corresponding EDCs. The data were measured at 30K, BL5-2 of SSRL.

6. Procedure for Fitting MDCs and self-energy analysis

(a) Fitting MDCs of ARPES spectra

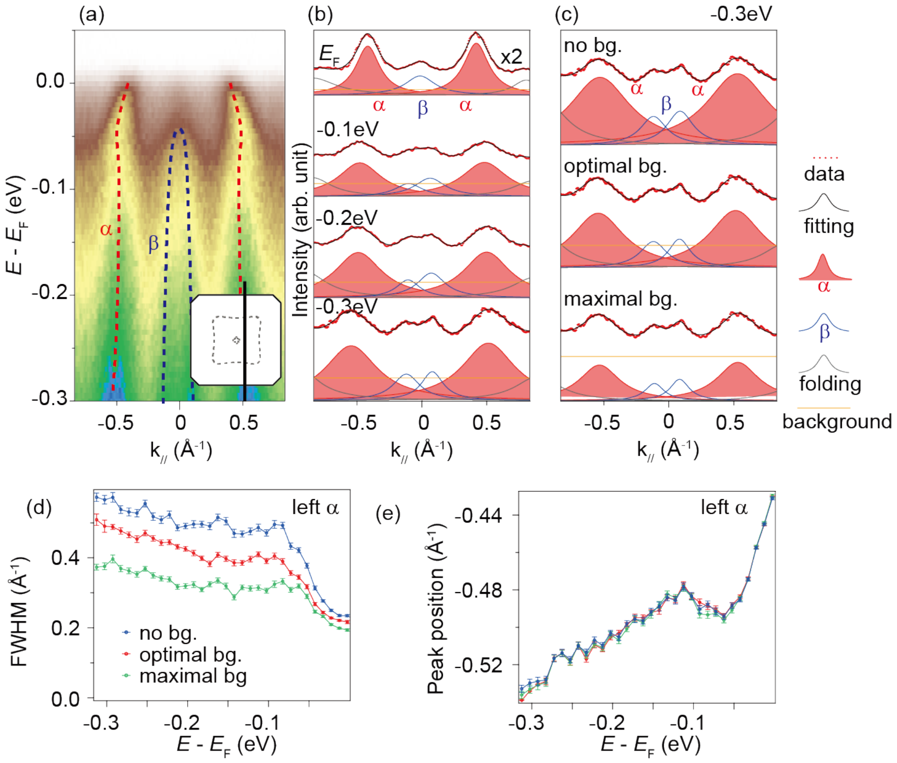

Supplementary Figure S8. (a) Photoemission spectra taken with p-polarized photons along the cut in the inset. (b) Multi-peak fittings of MDCs at representative binding energies. c) Example of fitting the MDC at -0.3eV with no background, optimal background, and maximal background. (c-d) WHM and peak position of the left branch of band alpha from fitting with three different background setups.

In order to extract the self-energy, we fit the MDCs of ARPES spectra to obtain the peak information of band α. As band β' crosses band α near the zone center and complicates the analysis, we use data taken slightly off the zone center (see inset of Fig.S8a) so that the band β' is far below the Fermi energy. As shown in the photoemission image (Fig.S8a), the two branches of band α are most intensive, while band β is weaker. At the X point there are faint intensity from the folding of band β, which are also present in parent compound (Ref. 1). Since the folding from Γ to X is not expected in the G-type AFM order with **Q** = (1/2,1/2,1/2), its origin has not been identified and was speculated to relate with possible surface reconstructions (Ref. 1).

Based on the observed band structure, we used six Lorentzian peaks to represent two branches of bands α, β, and the folded β band at the left and right X points, respectively (Fig. S8b). An energy-dependent constant background is added for each MDC. The fitting function is further broadened by a Gaussian of FWHM=0.005 $Å^{-1}$, which corresponds to the 0.1-degree angular resolution of the ARPES setup.

The constant background that is stronger at higher binding energies (Fig. S8b), which may bring

extra uncertainties. We estimate the effect of the constant background value by manually set it from zero to the maximal value that gives a reasonable fitting (see the results on MDC at E_B=0.3eV in Fig.S8c for example). The FWHM of band α is significantly varied by the constant background value (Fig.S8d). Nevertheless, the low-energy part of the FWHM are more robust against the constant background value (Fig.S8d), and consistently show a step-like feature corresponding to the imaginary self-energy ImΣ of the kink. Furthermore, the peak position of band α is insensitive to the constant background value (Fig.S8e), which supports the reliability of the extracted dispersions and $\mathrm{Re}\Sigma^{b}$. Based on both the Kramers-Kronig conjugation between $\mathrm{Re}\Sigma^{b}$ and $\mathrm{Im}\Sigma^{b}$, and the overall fitting quality, we estimated the optimal background value and extracted the self-energy of band α in the main text.

(b) Extraction of self-energy

The real part of the self-energy contributed by the kink-related bosonic mode is obtained by $\mathrm{Re}\,\Sigma^{\mathrm{b}}(E,\mathbf{k}) = E(\mathbf{k}) - E_0(\mathbf{k})$, where $E(\mathbf{k})$ and $E_0(\mathbf{k})$ are the renormalized energy and the "bare band" energy, respectively. The "bare band" is estimated by a parabola fit to the Fermi crossing and data above the kink energy.

The imaginary part of the self-energy can be extracted by $|\mathrm{Im}\,\Sigma(E,\mathbf{k})| = |v_{\mathrm{F}}^{0}| \times \frac{\mathrm{FWHM}}{2}$, where FWHM is the full-width at the half-maximum of the MDC peak and v_{F}^{0} is the "bare band" velocity. The imaginary part $\mathrm{Im}\,\Sigma(E,\mathbf{k})$ includes the contribution from the kink-related bosonic mode $\mathrm{Im}\,\Sigma^{\mathrm{b}}(E,\mathbf{k})$, and $\mathrm{Im}\,\Sigma^{\mathrm{others}}(E,\mathbf{k})$ including from extrinsic momentum broadening and other correlation effects. Our analysis mainly focuses on the part contributed by the bosons centered around 50meV, i.e. $\mathrm{Im}\,\Sigma^{\mathrm{b}}(E,\mathbf{k})$.

The uncertainty of the self-energy is given by:

$$\Delta\,\mathrm{Re}\,\Sigma^{\mathrm{b}}(E,\mathbf{k}) = |v_{\mathrm{F}}^{0}| \times \Delta\mathbf{k}$$

$$\Delta\,\mathrm{Im}\,\Sigma(E,\mathbf{k}) = |v_{\mathrm{F}}^{0}| \times \frac{\Delta\,\mathrm{FWHM}}{2}$$

Where $\Delta\mathbf{k}$ and ΔFWHM are from uncertainties of peak positions and FWHM obtained in the fitting process in Section 4, and further combined with momentum resolution corresponding to the 0.1-degree angular resolution of ARPES instrument.

(a) The Kramers-Kronig transition

The $\mathrm{Re}\,\Sigma^{\mathrm{b}}(E,\mathbf{k})$ and $\mathrm{Im}\,\Sigma^{\mathrm{b}}(E,\mathbf{k})$ should be the Kramers-Kronig conjugation of each other:

$$\mathrm{Re}\,\Sigma_{\mathrm{KK}}^{\mathrm{b}}(E,\mathbf{k}) = \frac{1}{\pi}\int_{-\infty}^{+\infty} \frac{\mathrm{Im}\,\Sigma^{b}(E,\mathbf{k})}{E'-E} dE'$$

$$\mathrm{Im}\,\Sigma_{\mathrm{KK}}^{\mathrm{b}}(E,\mathbf{k}) = -\frac{1}{\pi}\int_{-\infty}^{+\infty} \frac{\mathrm{Re}\,\Sigma^{b}(E,\mathbf{k})}{E'-E} dE'$$

where $\mathrm{Im}\,\Sigma^{\mathrm{b}}(E,\mathbf{k}) = \mathrm{Im}\,\Sigma(E,\mathbf{k}) - \mathrm{Im}\,\Sigma^{\mathrm{others}}(E,\mathbf{k})$

$\mathrm{Re}\,\Sigma^{\mathrm{b}}$ drops to zero above 250 meV binding energy in our data, indicating that the effect electron-boson interactions is saturated beyond this energy scale. We can safely assume the tail of $\mathrm{Re}\,\Sigma^{\mathrm{b}}$ to be zero and the tail of $\mathrm{Im}\,\Sigma^{\mathrm{b}}$ to be a constant as:

$$\mathrm{Re}\,\Sigma^{\mathrm{b}}_{\mathrm{KK}}(E,\mathbf{k}) = 0, E > 300\ \mathrm{meV}$$

$$\mathrm{Im}\,\Sigma^{\mathrm{b}}_{\mathrm{KK}}(E,\mathbf{k}) = \mathrm{Im}\,\Sigma^{\mathrm{b}}_{\mathrm{KK}}(300\ \mathrm{meV},\mathbf{k}), E > 300\ \mathrm{meV}$$

Since $\int_{-\infty}^{+\infty} \frac{c}{x'-x} dx' = 0$, where c is a constant, adding an arbitrary constant term would not change the result of the Kramers-Kronig transformation.

(c) Calculation of the coupling constant

The coupling constant λ can be estimated by two methods:

1) The renormalization of the Fermi velocity.

$$\lambda = v_0/v_{\mathrm{F}} - 1$$

2) The Eliashberg function from the self-energy.

In the quasi-elastic approximation where the electronic energy E is much larger than the boson energy ω, the quasiparticle self-energy is determined by the Eliashberg function:

$$\mathrm{Im}\,\Sigma^{\mathrm{b}}(E,\mathbf{k}) = \pi \int_0^{\omega_{\max}} \alpha^2 F(E,\mathbf{k};\omega)[1 - f(E-\omega) + f(E+\omega) + 2n(\omega)]d\omega$$

Assuming the $T \to 0$ limit with $n(\omega) \to 0$, we have:

$$\mathrm{Im}\,\Sigma^{\mathrm{b}}(E,\mathbf{k}) = \pi \int_0^{\omega_{\max}} \alpha^2 F(E,\mathbf{k};\omega) f(E+\omega) d\omega$$

As the electronic energy scale of band α is much larger than the magnon energy scale, the dependence of $\alpha^2 F(E,\mathbf{k};\omega)$ as a function of the electronic energy can be ignored. In this case, the variation in $\mathrm{Im}\,\Sigma(E,\mathbf{k})$ with E mainly comes from the change in the effective integration range due to the Fermi-Dirac function $f(E+\omega)$. Thus, we can estimate $\alpha^2 F(\omega)$ by taking the derivative of $\mathrm{Im}\,\Sigma^{\mathrm{b}}(E,\mathbf{k})$:

$$\alpha^2 F(\omega) = \frac{\partial \mathrm{Im}\,\Sigma^{\mathrm{b}}(E,\mathbf{k})}{\pi \partial E}\Big|_{E=\omega}$$

From the numerical data, the Eliashberg function is estimated as:

$$\alpha^2 F(E) = \frac{\Sigma^{\mathrm{b}}(E+\Delta E,\boldsymbol{k}) - \Sigma^{\mathrm{b}}(E-\Delta E,\boldsymbol{k})}{2\pi \Delta E}$$

where $\Delta E = 10$ meV represents the energy step in our data.

To avoid nonphysical values, we set the noise-induced negative data points to zero. The coupling constant λ can be estimated by:

$$\lambda = 2\int_0^{\omega_{\max}} \frac{\alpha^2 F(\omega)}{\omega} d\omega$$

For both cases, we employed the formula at the $T \to 0$ limit because the experimental temperatures were far below the T_N of $Ba_{1-x}K_xMn_2As_2$.

7. Spectral function simulation

We simulated the photoemission spectra of Fig.2(b) by:

$$I_{ARPES} = I_0 \times [A(E,k) \times F(E,k)] \otimes R(E,k) + B(E),$$

where $A(E,k) = -\frac{1}{\pi}\frac{\mathrm{Im}\,\Sigma^{raw}(E,\mathbf{k})}{[E-E_0(\mathbf{k})-\mathrm{Re}\,\Sigma^{b}(E,\mathbf{k})]^2+[\mathrm{Im}\,\Sigma^{raw}(E,\mathbf{k})]^2}$ is the quasiparticle spectral function, $R(E,k)$ is the Gaussian convolution of energy and momentum broadening, and $B(E)$ is the energy dependent constant background in MDC fittings.

The result of simulation (Fig.S9a) is compared side-by-side with the raw data (Fig.S9b). The simulated spectra show decreasing peak intensity at higher binding energies. The high-energy incoherent feature in the raw data does not show up in the simulation, which suggests that the high-energy incoherent feature is beyond the conventional quasiparticle picture. Similar high-energy incoherent features have been generally observed in correlated materials, including cuprates [4] and manganites [5].

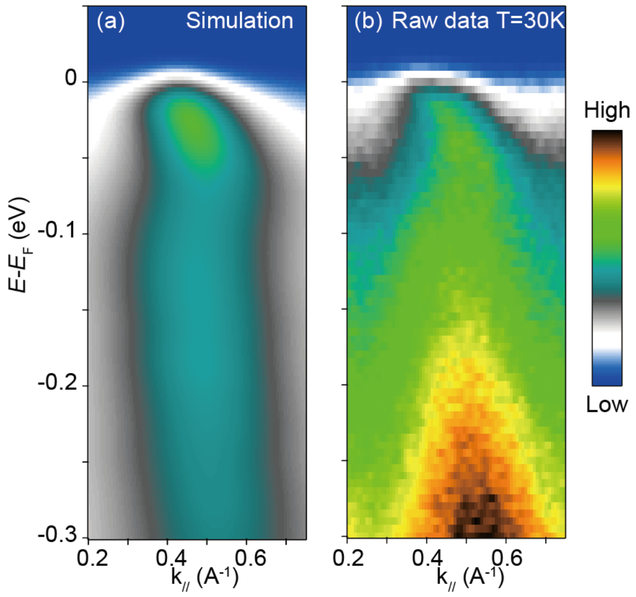

Supplementary Figure S9 (a) Simulated spectra based on the extracted self-energies and non-dispersive background. (b) Raw data of Fig.2b, from which the self-energies are extracted.

8. Contribution of the electron-electron correlations

As shown in Fig.2e of the main text, the imaginary part of self-energy extracted by the FWHM of MDC peaks ($\mathrm{Im}\,\Sigma^{raw}(E,\mathbf{k})$) contains a large background ($\mathrm{Im}\,\Sigma^{others}(E,\mathbf{k})$). The $\mathrm{Im}\,\Sigma^{others}(E,\mathbf{k})$ consists of a constant and a quadratic term. The constant term is likely contributed by extrinsic momentum broadening of instrumental resolution and sample surface imperfection. The quadratic term has negligible contribution to $\mathrm{Im}\,\Sigma^{raw}(E,\mathbf{k})$ at low-energy range (E_B<100meV) and mainly accounts for the high-energy rise in $\mathrm{Im}\,\Sigma^{raw}(E,\mathbf{k})$ at E_B>100meV.

Though the quadratic term is regularly considered to be from the electron-electron correlation in the form of Landau Fermi liquid behavior, a quantitative estimation of the contribution from the Fermi-liquid-like electron-electron correlation is not achievable based on the current data of $Ba_{1-x}K_xMn_2As_2$. First, in $Ba_{1-x}K_xMn_2As_2$, the large-scale dispersion of band α is mainly contributed by the incoherent weight from strong correlation, whose centroid generally follows the bare band dispersion [6]. Therefore, it is not reliable to conclude the strength of electron-electron correlation in $Ba_{1-x}K_xMn_2As_2$ based on the bandwidth. Second, the high-energy incoherent weight in $Ba_{1-x}K_xMn_2As_2$ deviates from a conventional Landau quasiparticle picture (see Section 6) and it dominates the high-energy spectra. Therefore, one cannot attribute the quadratic term of $\mathrm{Im}\,\Sigma^{others}(E,\mathbf{k})$ to the Fermi-liquid-like electron-electron correlation to estimate its strength quantitatively. Nevertheless, a moderate Fermi-liquid-like electron-electron correlation is possible in $Ba_{1-x}K_xMn_2As_2$, which contributes the large coupling constant together with the electron-magnon interactions.

Though we can neither exclude the presence of Fermi-liquid-like electron-electron correlation nor estimate its strength quantitatively in $Ba_{1-x}K_xMn_2As_2$, we could conclude that the kink is not induced by the purely electronic mechanism that does not relate to any coupling of excitations [7]. According to the theory of purely electronic kinks [7], the kink energy should be proportional to the electron-electron interacting strength. However, the kink energy of $Ba_{1-x}K_xMn_2As_2$ remains around 60 ± 10meV regardless of the varying coupling constant from 1.9 (x=0.1) to 5.4 (x=0.3) [Fig.3(g)], which does not support the pure electronic mechanism of kinks.

9. Rigid band behavior of band α

To see the rigid band shift, here we compared the spectra of different dopings at the full energy scale of band α. As shown in Fig. S10, besides the shifting of chemical potential by doping, the dispersions of band α for x=0.2 and 0.3 perfectly follow that of x = 0.1 within experimental resolution, demonstrating a rigid band behavior.

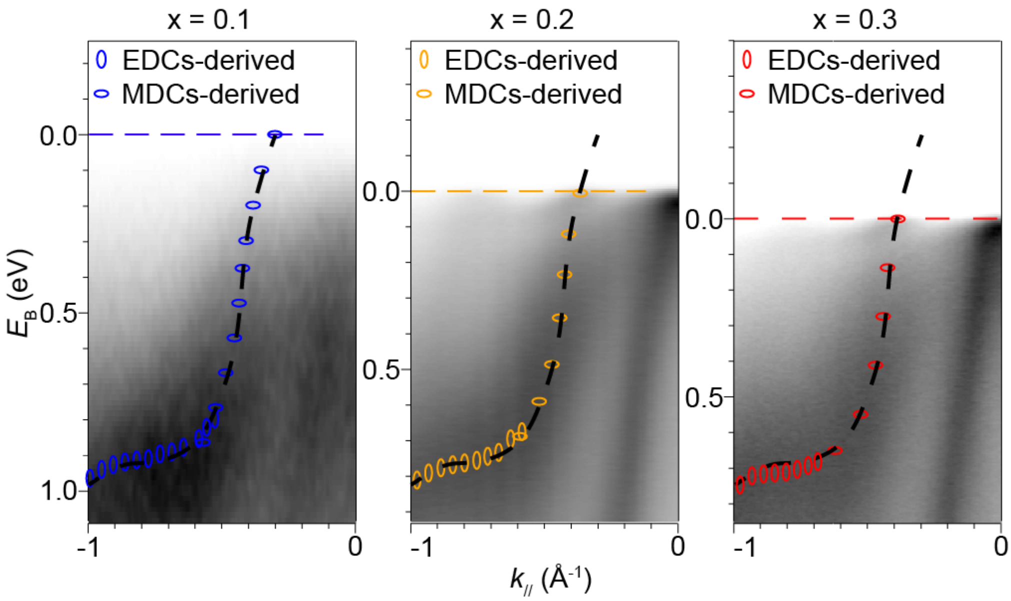

Supplementary Figure S10. Large scale dispersion of band α for x = 0.1 (a), 0.2 (b) and 0.3 (c) from MDCs and EDCs. The data were measured at 30K, BL5-2 of SSRL.

10. Procedures for estimating the DOS at E_F

The DOS at E_F, $N(E_F)$, is estimated by the following equations based on its definition:

$$N(E_\mathrm{F}) = \frac{1}{2} \times \frac{\pi}{2} \int \frac{dS}{4\pi^3} \frac{1}{|\nabla E_n(\mathbf{k})|} \sim \frac{V_0 S_\mathrm{FS}}{16\pi^3} \overline{|v_\mathrm{F}^{-1}|}$$

Where V_0 is the volume of the unit cell, S_{FS} is the Fermi surface area and v_F is the Fermi velocity. The "bare band" density of states $N^0(E_F)$ are obtained in the same way by replacing the Fermi velocity v_F with the "bare band" velocity v_F^0.

We estimate the S_{FS} of the drum-shaped Fermi pocket α by an analogue of the body-centered tetragonal BZ, a truncated hexahedron, and the S_{FS} of band β/β' based on elliptical cylinders. The uncertainty of the S_{FS} is estimated to be 15% (see Section 2). The $\overline{|v_\mathrm{F}^{-1}|}$ calculation is based on the measured value of v_F and the assumption of a linear momentum dependence. Thus, $\overline{|v_\mathrm{F}^{-1}|} = \frac{\int_{v_\mathrm{Fmin}}^{v_\mathrm{Fmax}} v_\mathrm{F}^{-1} dv_F}{v_\mathrm{Fmax} - v_\mathrm{Fmin}} = \frac{\ln(v_\mathrm{Fmax}) - \ln(v_\mathrm{Fmin})}{v_\mathrm{Fmax} - v_\mathrm{Fmin}}$.

11. Calculated phonon spectra

The calculated phonon density mainly locates below 25 meV for both $x = 0$ and $x = 0.5$. The calculations were performed by using the VASP [8,9] and Phonopy [10] code. The projected-augmented wave (PAW) pseudopotentials with Perdew-Burke-Ernzerhof (PBE) form ref. 11 of exchange-correlation functional are employed. Phonon dispersion calculations were performed based on the finite difference method. The $\sqrt{2} \times \sqrt{2} \times 1$ $BaMn_2As_2$ supercells with displacements are used for the force constants calculations. The lattice parameters are $a = 4.359$ Å and $c = 13.487$ Å. For potassium doped compound of $Ba_{0.5}K_{0.5}Mn_2As_2$, one of every two Ba atoms is replaced by one K atom. The plane wave cutoff energy of 500 eV, an energy convergence criterion of 10^{-6} eV and a 4×4×4 Monkhorst-Pack k-point mesh were set.

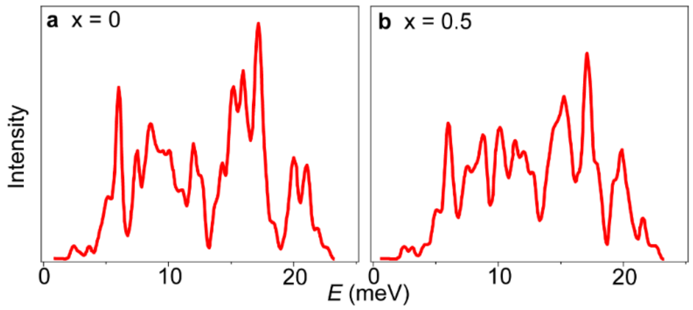

Supplementary Figure S11. Calculated phonon spectra for different dopings: $x = 0$ (a) and $x = 0.5$ (b).

12. Simulation of magnon spectra

The magnon spectra of $Ba_{1-x}K_xMn_2As_2$ have been studied by inelastic neutron scattering (INS) measurements in previous reports [12]. The dispersive spectra from INS demonstrates that the magnon spectral intensity of AFM order in (Ba,K)Mn_2As_2 is distributed over the reciprocal space. The magnon spectra of INS have been well reproduced by simulations based on Heisenberg model [12]. Here we calculated the magnon dispersion $\omega(\boldsymbol{q})$ and intensity $I(\boldsymbol{q},\omega)$ of $Ba_{1-x}K_xMn_2As_2$ x=0.1, 0.2, and 0.3 following the model in ref. 12 by the SpinW package [13]. The calculation was performed in a 1-Mn magnetic Brillouin zone. The magnetic exchange interaction J_1, J_2, and J_c were obtained by interpolating the doping dependent values in Ref. 12 from fitting the INS data. The results along the high symmetric momentum scan are shown in Fig. S12(a).

The total density of states (DOS) of magnons was also obtained from the spin wave calculation by $\mathrm{DOS}(\omega) = \iiint I(\boldsymbol{q},\omega)d\boldsymbol{q}$. The total DOS shows a systematic shift towards lower energy as the doping increases, which is consistent with the INS results [12]. Though the total magnon DOS deviates from the magnetic intensity of INS in line-shape due to the energy-dependent kinematic cutoff on the low-Q side of the detector, they are consistent in energy scale (Fig. S12c).

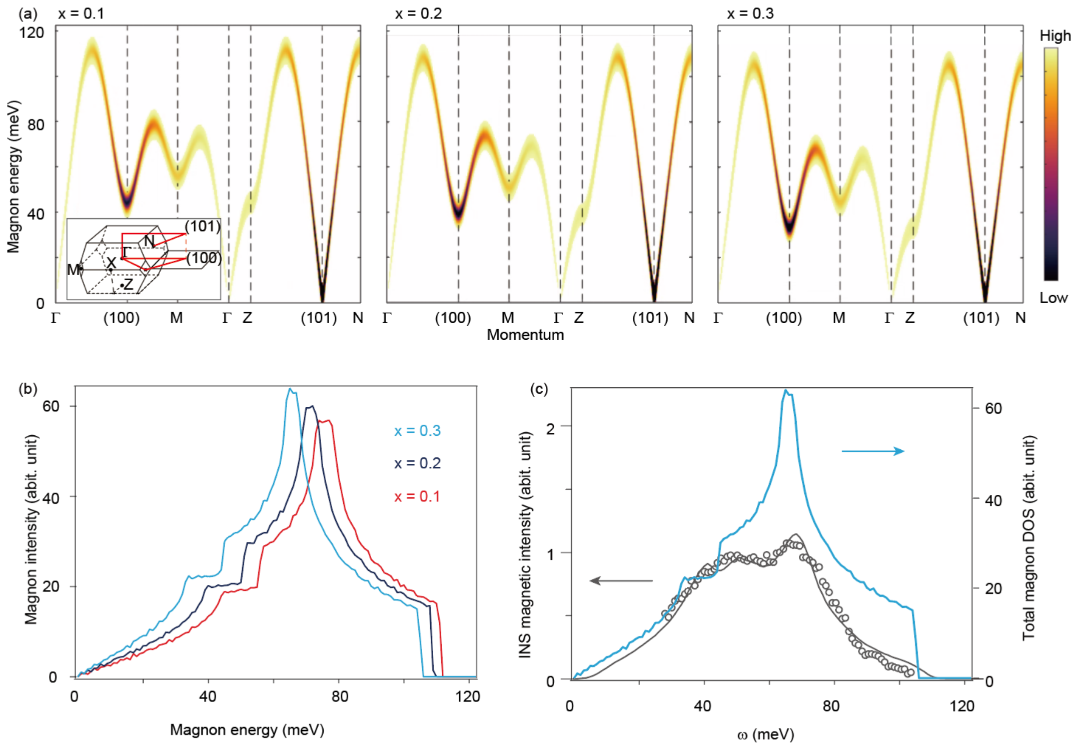

Supplementary Figure S12. (a) Calculated magnon dispersion and intensity of $Ba_{1-x}K_xMn_2As_2$ x=0.1, 0.2, and 0.3. The inset illustrates the momentum scan of the calculation. (b) Total magnon density of states obtained by integrating the magnon intensity over the reciprocal space. (c) Measured (gray circles) and simulated (gray curve) magnetic intensity of inelastic neutron scattering from ref. 12, compared with our calculated total magnon DOS (blue curve).

13. Simulation of magnon's contribution to the Eliashberg function $\alpha^2F(\omega)$

As the magnon spectra are more dispersive than those of regular optical phonons, the momentum conservation may restrict only a subset of the magnons to participate in the scattering for each electron momentum, under the approximation that the propagating electron/hole emits and absorbs a single magnon (this approximation is generally used in the photon emission/absorption in electron-electron correlation as the GW approximation and the phonon emission/absorption in electron-phonon interaction as Migdal approximation, as sketched in Fig. S13b).

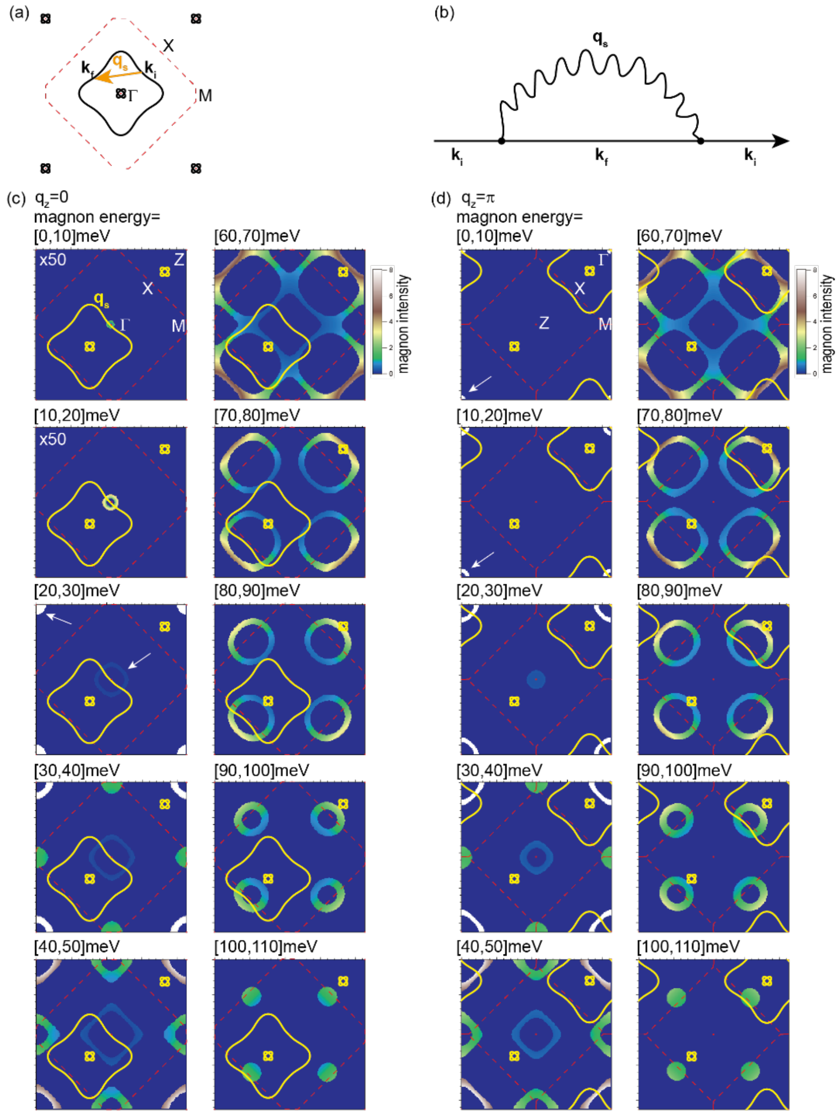

Supplementary Figure S13. (a) Illustration of the k_z=0 Brillouin zone (dashed red lines), Fermi surfaces (black curves), and the scattering vector q_s between electronic states (orange arrow). (b) Diagram illustrating the process of emitting and absorbing a single magnon of momentum $q_s = k_f - k_i$. (c) magnon intensity in the q_z=0 plane and 10meV energy windows. The allowed scattering vectors q_s are illustrated as yellow curves. (d) same as (c) but in the q_z=π plane.

Following the approximation of single magnon scattering process, we simulated the spin wave's contribution near the Fermi momentum along the ΓX cut ($\boldsymbol{k_i}$ in Fig. S13a) for $Ba_{1-x}K_xMn_2As_2$ x=0.3. Following the quasi-elastic approximation, the allowed scattering vectors $\boldsymbol{q_s}$ are determined by:

$$\boldsymbol{q_s}(\boldsymbol{k_f}, \boldsymbol{k}_i) = \boldsymbol{k}_f - \boldsymbol{k}_i$$

where $\boldsymbol{k_f}$ goes through the Fermi crossings over the 3-dimensional Brillouin zone. For visualizing the magnons' contribution to the $\alpha^2F(\omega)$, we take the planes of $q_z = 0$ and $q_z = \pi$ in the reciprocal space for illustration purpose. The magnon intensity are plotted in 10meV energy windows for the $q_z = 0$ plane (Fig. S13c) and $q_z = \pi$ plane (Fig. S13d), respectively. The intensity in each panel is set as

$$I(q_x, q_y, \omega) = \begin{cases} I(q_x, q_y, q_z, \omega), & for\ \omega\ in\ the\ 10meV\ window, and\ q_z = 0\ or\ \pi; \\ 0, & for\ \omega\ outside\ the\ 10meV\ window. \end{cases}$$

The allowed scattering vectors $\boldsymbol{q_s}$ are overlaid with the calculated magnon intensities as yellow curves. For every energy window, the magnon iso-energy surface (of 10meV energy window) crosses with the scattering vectors $\boldsymbol{q_s}$, indicating allowed electron-magnon scattering all over the [0, 110meV] energy range of magnon spectra.

The Eliashberg function is given by:

$$\alpha^2F(\epsilon_i, \boldsymbol{k}_i; \omega) = \sum_{\boldsymbol{q},f} \delta(\epsilon_i - \epsilon_f \pm \omega_{\boldsymbol{q}}) g^{i,f}(\boldsymbol{k}_i, \boldsymbol{q}) \delta(\omega - \omega_{\boldsymbol{q}})$$

where $g^{i,f}(\boldsymbol{k}, \boldsymbol{q})$ is the electron-magnon interaction matrix element. The first delta function restricts the scattering between states on electron bands, i.e., limited to $\boldsymbol{q_s} = \boldsymbol{k}_f - \boldsymbol{k}_i$. The second delta function takes the magnon dispersion and intensity into account. The Eliashberg function $\alpha^2F(\omega)$ could be viewed as an electron-electron correlation function weighted by magnons at $\boldsymbol{q_s}$ and $g^{i,f}(\boldsymbol{k}, \boldsymbol{q})$. As illustrated in Fig. S13, for every momentum where the magnon iso-energy surface and $\boldsymbol{q_s}$ overlap with each other, the electron-magnon scattering is allowed and the corresponding magnon intensity at these energies and momenta will contribute to the electron-magnon interaction and Eliashberg function. Assuming a constant *g*, the Eliashberg function at the specific electronic momentum $\boldsymbol{k}_i$ can be calculated by:

$$\alpha^2F(\boldsymbol{k}_i, \omega) = g \iiint \oiint I(\boldsymbol{q}, \omega) \cdot \delta(\boldsymbol{q} - \boldsymbol{q_s}(\boldsymbol{k_f}, \boldsymbol{k}_i)) \mathrm{d}\boldsymbol{q} \mathrm{d}\boldsymbol{k_f}$$

where the integrations of $\boldsymbol{q}$ and $\boldsymbol{k_f}$ go over the 3-dimensional Brillouin zone and the electronic Fermi surface, respectively. In our calculation, these integrations were performed by numerical summations over a 428×428×94 k-point mesh in the 1-Mn Brillouin zone. The Eliashberg function calculated in this way is plotted in Fig. S14, which is in arbitrary intensity as the magnitude of *g* is unknown. The energy range of the simulated Eliashberg function $\alpha^2F(\omega)$ fits that from photoemission data (Fig. S14b). Furthermore, both experimental and simulated $\alpha^2F(\omega)$ show the same energy scale as the total magnon DOS and magnetic intensity from INS (Fig. S14). This indicates that a large fraction of the magnon excitations participates the scattering with electrons, even under the constraints imposed by the Fermi surface and energy and momentum conservations.

Besides the consistent energy scale, we see a clear discrepancy in line-shape between the simulated magnon contribution and the $\alpha^2F(\omega)$ from ARPES, especially over the energy range around 20~60 meV (Fig. S14). This indicates that other factors beyond the above simple model of single magnon process may play a role in the line-shape, e.g., the momentum and energy dependencies of *g* or the higher order multi-magnon processes (which is likely dominating considering the large coupling constant λ). These observations constrain the future theories on the EAIs.

We conducted the simulation on electron-magnon coupling for different Fermi surface volumes of *x*=0.1, 0.2, and 0.3, using the approximation of single magnon scattering process. The overall

intensity of simulated α^2F increases with increasing doping (Fig. S15), demonstrating that the Eliashberg function is indeed enlarged for a larger Fermi surface.

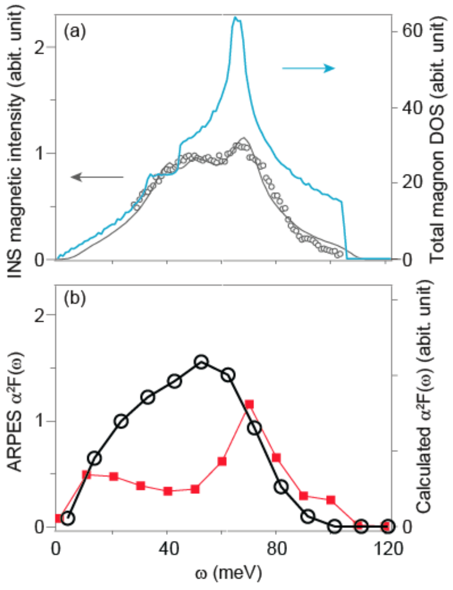

Supplementary Figure S14. (a) Measured (gray circles) and simulated (gray curve) magnetic intensity of inelastic neutron scattering from ref. 12, compared with our calculated total magnon DOS (blue curve). (b) Eliashberg function $\alpha^2F(\omega)$ from ARPES data (black circles) and $\alpha^2F(\omega)$ simulated at the k_F along ΓX (red squares, in arbitrary unit as the magnitude of g is unknown during calculation).

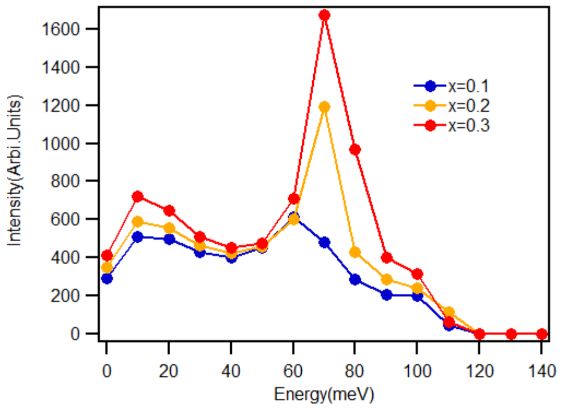

Supplementary Figure S15. Simulated α^2F for x = 0.1, 0.2 and 0.3.

14. Magnetization properties of $Ba_{1-x}K_xMn_2As_2$ (x = 0.3)

The magnetic susceptibility of $Ba_{1-x}K_xMn_2As_2$ (x = 0.3) shows an upturn below 100K, indicating a ferromagnetic transition (Fig. S16a-b). The *M-H* curve shows small coercive field (Fig. S16c), similar to the behavior of other itinerant ferromagnets. The saturated ferromagnetic magnetization is around 0.27 μ_B/f.u..

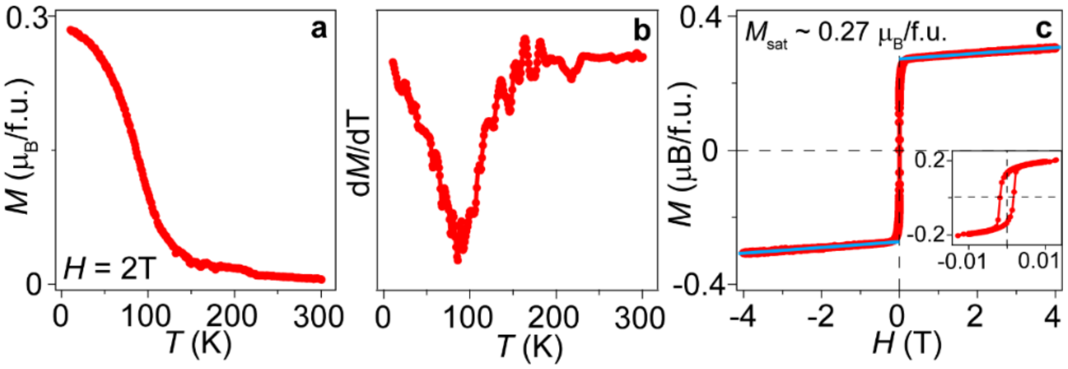

Supplementary Figure S16. (a) *M-T* curve at *H* = 2 T. (b) Derivative of the *M-T* curve with respect to the temperature. (c) *M-H* curves at 10 K.

15. The first principles spin-resolved calculation

First-principles density functional theory (DFT) calculations were performed by using the projector-augmented wave (PAW) method as implemented in the VASP code [9,14,15]. The exchange-correlation potential was calculated using the generalized gradient approximation (GGA) as proposed by Pedrew, Burke, and Ernzerhof [11]. The plane-wave cutoff energy and the convergence of total energy are set as 500 eV and 10^{-6} eV, respectively. The Γ-centered *k*-point mesh in the Brillouin zone is set as 8 × 8 × 4. For 2 × 2 × 1 supercell with 40 atoms, the lattice constant *a* and *c* are 8.30 Å and 13.47 Å, respectively. G-type antiferromagnetic (AF) configuration is adopted. Under this configuration, the Mn atoms have the opposite spin orientations with their nearest neighbors in the same plane and the corresponding Mn atoms on the other plane. In order to compare with the electronic structure of primitive cell which contains 5 atoms, band unfolding calculation are done with effective band structure (EBS) model [16,17] implemented in the VASPKIT code [18].

In the first-principle calculation by DFT+U method, we try to induce FM state by adding exchange interaction U at the As-4*p* orbital. However, with U up to 6 eV, the local moment on As-4*p* orbital is as small as 0.00067 μ_B. Therefore, both our experimental observation and DFT calculation suggest that the ferromagnetism in $Ba_{1-x}K_xMn_2As_2$ cannot be accounted for by a regular exchange splitting of itinerant bands. This is not surprising as the renormalization of electron-magnon interaction is not included in the framework of DFT calculations.

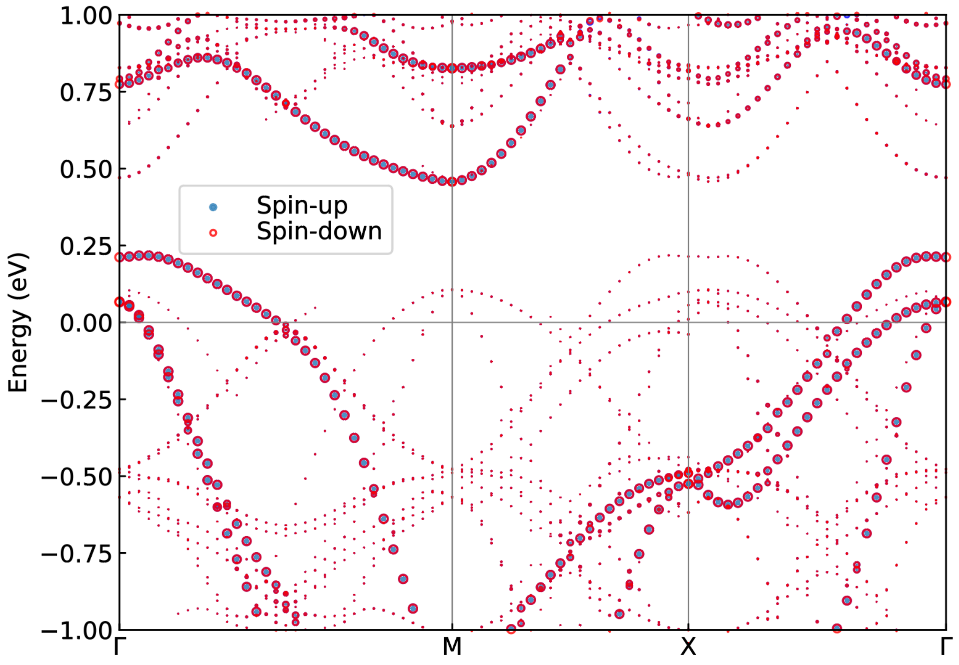

Supplementary Figure S17. The spin-resolved DFT calculation on $Ba_{0.75}K_{0.25}Mn_2As_2$. No clear splitting is found with 0~6 eV of U applied on As 4*p*. The thin spots are shadow bands of the 4-uc superlattice for introducing the K doping of *x*=0.25. The spin-up bands (blue filled circles) and spin-down bands (red empty circles) overlap with each other, indicating negligible exchange splitting in the framework of DFT calculations.

16. Possible ferromagnetic exchange splitting of the band α

In the out-of-plane Fermi surface map (Figs. S18a-b), the FWHM of MDCs increase by approximately 35% along k_z, while in the in-plane Fermi surface map (Fig. S18c-d), the FWMH of MDCs at E_F is approximately the same at different k_F. Without clear exchange splitting, the larger FWHM at k_z away from the ΓXM plane (Fig. S18e) is likely due to that the photoemission cut is closer to the tangent of the Fermi surface at the crossing point. Nevertheless, if we assume the presence of an exchange splitting, we could roughly estimate the upper limit of the exchange splitting by a double peak fitting of the band α at E_F (Fig.S18f). Based on the estimated possible splitting, the upper limit of the spin polarization is estimated to be ~0.1~holes/f.u. (f.u. = formula unit) based on the possible momentum splitting over the three-dimensional Fermi surfaces, which accounts for only 40% of the ferromagnetic moment of 0.25~μ_B/f.u. at 30~K by magnetic susceptibility measurements. It could be a rough estimation, but adequate to provide an upper limit of itinerant component in the emergent ferromagnetism in $Ba_{1-x}K_xMn_2As_2$. We emphasize that the double peak fitting does not support the presence of a splitting. We may tell the resolving of two peaks by whether there is a minimum at the midpoint of peaks, which is the Sparrow criterion FWHM/√3≈0.12Å^{-1} much larger than the Δk≈0.065 Å^{-1} from our double peak fitting (Figs.S18g-h). Therefore, the experimental data of band α is single-peak.

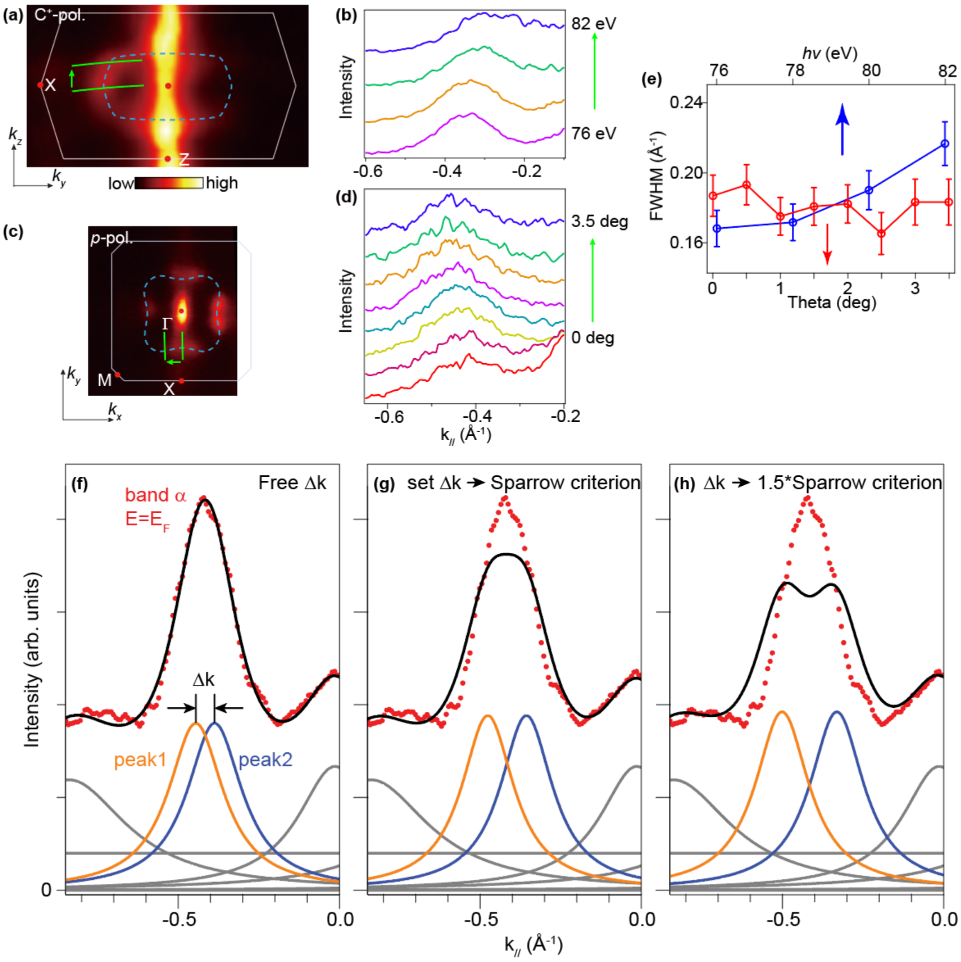

Supplementary Figure S18 (a) The out-of-plane Fermi surface map. (b) The MDCs from *hν* = 76 ~ 82 eV near band α at E_F. (c) The in-plane Fermi surface map. (d) The MDCs from theta = 0 ~ 3.5 degree at E_F. (e) The FWHM of band α at different *hν* and different emission angles, derived from panels e and d. (f-g) Two-peak fittings to the MDC of band α at E_F taken at *hν* = 78 eV, while the background and other bands are fixed to the single-peak fitting results. (f) Without any constrains, the double peak fitting gives the momentum splitting of k = 0.065 $Å^{-1}$. (g) Two-peak fitting by setting the Δk to the value of Sparrow criterion. (h) Two-peak fitting by setting the Δk to 1.5 times the value of Sparrow criterion. The data were measured at 30K, BL5-2 of SSRL.

References:

1. Zhang, W.-L. et al. Angle-resolved photoemission observation of Mn-pnictide hybridization and negligible band structure renormalization in $BaMn_2As_2$ and $BaMn_2Sb_2$. Phys. Rev. B **94**, 155155 (2016).
2. Shan, L. et.al., Observation of Ordered Vortices with Andreev Bound States in $Ba_{0.6}K_{0.4}Fe_2As_2$, Nature Physics, **7**, 325 (2011).
3. Lamsal, J., et. al., Persistence of Local-Moment Antiferromagnetic Order in $Ba_{1-x}K_xMn_2As_2$. Physical Review B, **87**, 144418 (2013).
4. Okawa, M. et al. Superconducting electronic state in optimally doped $YBa_2Cu_3O_{7-\delta}$ observed with laser-excited angle-resolved photoemission spectroscopy. Phys. Rev. B 79, 144528 (2009).
5. Mannella, N. et al. Nodal quasiparticle in pseudogapped colossal magnetoresistive manganites. Nature 438, 474–478 (2005).
6. Dessau, D. S. et al. k-Dependent Electronic Structure, a Large ``Ghost'' Fermi Surface, and a Pseudogap in a Layered Magnetoresistive Oxide. Phys. Rev. Lett. 81, 192–195 (1998).
7. Byczuk, K. et al. Kinks in the dispersion of strongly correlated electrons. Nature Phys 3, 168–171 (2007).
8. Kresse, G. & Furthmüller, J. Efficiency of ab-initio total energy calculations for metals and semiconductors using a plane-wave basis set, *Comput. Mater. Sci.* **6**, 15 (1996).
9. Kresse, G. & Furthmüller, J. Efficient iterative schemes for ab initio total-energy calculations using a plane-wave basis set, *Phys. Rev. B* **54**, 11169 (1996).
10. Togo, A & Tanaka, I. First principles phonon calculations in materials science, *Scripta Materialia*, **108**, 1 (2015).
11. Perdew, J. P., Burke, K. & Ernzerhof, M. Generalized Gradient Approximation Made Simple, *Phys. Rev. Lett.* **77**, 3865 (1996).
12. Ramazanoglu, M. et.al., Robust Antiferromagnetic Spin Waves across the Metal-Insulator Transition in Hole-Doped $BaMn_2As_2$, *Physical Review B* **95**, 224401 (2017)
13. Toth, S. & Lake, B. Linear spin wave theory for single-Q incommensurate magnetic structures, *J. Phys.: Condens. Matter* **27**, 166002 (2015).
14. Blöchl, P. E. Projector augmented-wave method, *Phys. Rev. B* **50**, 17953 (1994).
15. Kresse, G. & Joubert, D. From ultrasoft pseudopotentials to the projector augmented-wave method, *Phys. Rev. B* **59**, 1758 (1999).
16. Popescu, V. & Zunger, A. Effective Band Structure of Random Alloys, *Phys. Rev. Lett.* **104**, 236403 (2010).
17. Popescu, V. & Zunger, A. Extracting E versus k effective band structure from supercell calculations on alloys and impurities, *Phys. Rev. B* **85**, 085201 (2012).
18. Wang, V., *et al.* VASPKIT: A User-Friendly Interface Facilitating High-Throughput Computing and Analysis Using VASP Code, *Computer Physics Communications* **267**, 108033 (2021).
